# A quadruplex real-time PCR assay combined with a conventional PCR for the differential detection of Marek’s disease virus vaccines and field strains

**DOI:** 10.3389/fvets.2023.1161441

**Published:** 2023-05-12

**Authors:** Shaopeng Wu, Tian Ding, Hongxia Shao, Kun Qian, Jianqiang Ye, Aijian Qin

**Affiliations:** ^1^Ministry of Education Key Laboratory for Avian Preventive Medicine, Yangzhou University, Yangzhou, Jiangsu, China; ^2^Jiangsu Co-Innovation Center for Prevention and Control of Important Animal Infectious Diseases and Zoonoses, Yangzhou University, Yangzhou, Jiangsu, China; ^3^Joint International Research Laboratory of Agriculture and Agri-Product Safety, The Ministry of Education of China, Yangzhou University, Yangzhou, Jiangsu, China

**Keywords:** Marek’s disease virus, vaccination, CVI988/Rispens, real-time PCR, detection

## Abstract

To evaluate the effect of the vaccine and differentiate vaccine from virulent MDV, a new quadruplex real-time PCR assay based on TaqMan probes was developed to differentiate and accurately quantify HVT, CVI988 and virulent MDV-1. The results showed that the limit of detection (LOD) of the new assay was 10 copies with correlation coefficients >0.994 of CVI988, HVT and virulent MDV DNA molecules without cross-reactivity with other avian disease viruses. The intra-assay and inter-assay coefficients of variation (CVs) of Ct values for the new assay were less than 3%. Analysis of replication kinetics of CVI988 and virulent MDV of collected feathers between 7 and 60 days post-infection (dpi) showed MD5 had no significant effect on the genomic load of CVI988 (*p* > 0.05), while vaccination with CVI988 could significantly reduce the viral load of MD5 (*p* < 0.05). Combined with *meq* gene PCR, this method can effectively identify virulent MDV infections in immunized chickens. These results demonstrated that this assay could distinguish between the vaccine and virulent MDV strains and had the advantages of being reliable, sensitive and specific to confirm the immunization status and monitor the circulation of virulent MDV strains.

## Introduction

Marek’s disease (MD) is a very important immunosuppressive and lymphoid tumor disease in poultry industry, which is caused by an α-herpesvirus, Gallid herpesvirus 2 (GaHV-2), traditionally known as Marek’s disease virus (MDV). According to the report of the International Committee on Taxonomy of Viruses (2019), GaHV-2, GaHV-3 and Meleagrid alphaherpesvirus 1 (MeHV-1 or Herpesvirus of Turkey, HVT) are all members of the genus Mardivirus of Alphaherpesvirinae subfamily (1). Among of them, only GaHV-2 causes neoplastic disease in chickens (2), while GaHV-3 and HVT are naturally avirulent (3). Since HVT vaccine was launched in the 1970s, the bivalent HVT + SB1 vaccine had been developed in the late 1980s, and the CVI988 vaccine had been used in the 1990s (USA) and 1970s (Europe) (4). In addition, the *meq* gene is only present in GaHV-2 but not GaHV-3 and MeHV-1. The virulent MDV strain deleted *meq* gene completely loses the ability to induce tumors, suggesting a key role for the *meq* gene in tumorigenesis (5). Therefore, many research groups have constructed *meq*-deleted recombinant MDV, which has proven to be vaccine candidates. For instance, the recombinant virus rMd5∆*meq* and SC9-1 can be used as vaccines to effectively prevent challenge with very virulent MDV (6–8). These vaccines have achieved great success in MD control. However, the vaccines can reduce virus-induced tumors and mortality but they do not prevent the infection and shedding of viruses (9, 10), which means chickens can be potentially infected with both vaccine and virulent MDV simultaneously.

The main methods currently used for the detection of MDV infection include virus isolation in CEF, the immunodiffusion test, PCR and real-time PCR. To distinguish CVI988 from virulent MDVs, molecular diagnostic methods have been developed based on the several types of different sequences such as polynucleotide sequence expansions, short tandem repeat polymorphisms (132-bp repeat region) (11), gene length variations (180-bp insertion of duplicated sequence in the *meq* gene of CVI988) (12), frameshift mutations and single nucleotide polymorphisms (SNPs) (13). Based on a stable polymorphism in the *meq* gene, Renz et al. developed two sensitive and specific real-time PCRs to differentiate and accurately quantify 20 virulent MDV isolates and 3 commercial CVI988 vaccines used in Australia (14). Baigent et al. designed and optimized a real-time PCR assay based on SNP #320 in the *pp38* gene to specifically distinguish CVI988 from virulent MDV-1 strains such as MD5, GA and RB1B (15, 16).

As a widely used vaccine vector, HVT has been applied to control several avian diseases, including avian influenza (AI) (17, 18) and infectious bursal disease (IBD) (19) by encoding heterologous antigen proteins as dual or triple vaccines (20). Compared to distinguishing CVI988 from virulent MDV, HVT is easier to identify due to the low degree of genomic homology (21–26). Usually, we could distinguish MDV vaccine from the field MDV strain by sizes of *meq* gene. However, we found that *Smeq* was also present in the CVI988 vaccine. In addition, we also found some vaccines such as 814 strain (GenBank accession number: JF742597.1), which is widely used in China (27–29), and has the same SNPs of *pp38* gene as virulent MDV, make it difficult to distinguish vaccines from field strains by real-time PCR alone.

In this study, a quadruplex real-time PCR assay was developed based on SNP #320 and #326 in the *pp38* gene of MDV-1 and *sorf1* gene of HVT to specifically distinguish CVI988, HVT, and virulent MDV-1 strains in one reaction. Combined with *meq* gene PCR, this method can effectively identify virulent MDV infections in immunized chickens. The specificity, sensitivity, repeatability, and reproducibility of the assay were evaluated. Moreover, the new assay can be used to detect the clinical samples and assess the viral replication dynamics of virulent and vaccine viruses in chicken feather tips.

## Materials and methods

### Viruses and samples

All viruses used in this study were stored in the Ministry of Education Key Lab for Avian Preventive Medicine, Yangzhou University, including the very virulent strain MD5 and RB1B [from Avian Disease and Oncology Laboratory (ADOL)], CVI988/Rispens (from Boehringer Ingelheim company), HVT and other avian disease viruses including Avian leukosis virus (ALV), Reticuloendotheliosis virus (REV), Chicken anemia virus (CIAV), Avian influenza virus (AIV), Newcastle disease virus (NDV), Fowl adenovirus (FAV) and Infectious bursal disease virus (IBDV). All MDV strains were prepared in chicken embryo fibroblast cells (CEF). These MD5-infected CEFs (at passage 4) and CVI988-infected CEFs (at passage 6) were used for the challenge and vaccination in animal experiment. ALV, REV, IBDV, NDV and AIV were cultured in DF-1 cells. Total RNA was extracted by FastPure Cell/Tissue Total RNA Isolation Kit (Vazyme Biotech Co., Ltd., Nanjing, China). Reverse transcription of RNA to cDNA was conducted using the PrimeScript RT reagent Kit (TaKaRa, Dalian, China) following the manufacturer’s instruction. FAV and CIAV were prepared in LMH and MSB1 cells, respectively. DNA was extracted by Axyprep Multisource Total DNA Miniprep Kit (Axygen, Hangzhou, China). The eluted nucleic acids were stored at −80°C. A total of 112 feather samples were submitted to our laboratory from 5 flocks located in four cities (Tangshan from Hebei province, Dalian from Liaoning province, Yinchuan from Ningxia province, Lishui from Zhejiang province) of China.

### Primers and probes

To establish the quadruplex real-time PCR assay, a pair of primers and two probes were designed in the highly conserved and unique regions in the *pp38* gene of CVI988 and virulent MDV-1 strains according to the previous report (15). A pair of primers and a probe for the detection of HVT were designed in the highly conserved regions of the *sorf1* gene in HVT genome. In addition, the primers and probe for detection of the chicken *ovotransferrin* (*ovo*) gene were reported previously (30). The four types of TaqMan probes were labeled with different fluorophores (FAM, Red610, Cy5, and Vic) at the 5′ end. All primers and probes were synthesized by BGI (Shanghai, China) and displayed in Table 1.

**Table 1:** Primer and probe sequences of quadruplex real-time PCR assay and PCRs.

Assay	Target		Probe/primer sequence	Position	Genes	Product size (bp)	References
Quadruplex real-time PCR	CVI988-pp38	F	GAGCTAACCGGAGAGGGAGA	289–308	pp38	99	15
		R	CGCATACCGACTTTCGTCAA	368–387			
		P	FAM-CCCACCGTGACAGCC-BHQ1	311–325			
	VIR-pp38	F	GAGCTAACCGGAGAGGGAGA	289–308	pp38	99	15
		R	CGCATACCGACTTTCGTCAA	368–387			
		P	Red610-CTCCCACTGTGACAGCC-BHQ2	311–327			
	HVT-sorf1	F	AACGTACGTCCAAGCAAGCG	484–503	sorf1	99	This study
		R	GTTCCCTCGTTCAGGTTGGC	563–582			
		P	Cy5-TCACGTACAGTCCCGCGTCTGTCGGTT-BHQ2	528–554			
	ovo	F	CACTGCCACTGGGCTCTGT	4517–4535	ovo	71	31
		R	GCAATGGCAATAAACCTCCAA	4567–4587			
		P	Rox-AGTCTGGAGAAGTCTGTGCAGCCTCCA-BHQ2	4537–4563			
Conventional PCR	meq	F	ATGTCTCAGGAGCCAGAGCC	1–20	meq	1020/1197	This study
		R	TCAGGGTCTCCCGTCACCT	1178–1197/1001–1020			

F, P, R indicate forward primer, probe, and reverse primer, respectively.

### Standard curve

To prepare the standard positive controls, the *pp38-RB1B*, *pp38-CVI988*, *sorf1-HVT* and *ovo* genes were cloned into the pGEM-T vector and transformed into DH5α chemically competent cells. Clones with the correct insert were confirmed by sequencing by Sangon Biotech (Shanghai, China). The concentration of vectors above was converted into copy numbers using the following formula: y (copies/μL) = (6.02 × 10^23^) × (x (ng/μL) × 10^−9^ DNA)/(DNA length × 660). The plasmid was diluted with ddH_2_O to obtain a stock solution containing 10^9^ copies of the standard plasmid per microliter. The standard curve was generated using 10-fold dilutions (10^3^–10^9^ copies/μL) of the standard plasmid.

### Development and optimization of the quadruplex real-time PCR

The quadruplex real-time PCR assay was performed using Premix Ex Taq (Probe qPCR, 2×) (TaKaRa) and the LightCycler^©^96 (Roche, Basel, Switzerland). The concentrations of primers and probes were optimized to yield the lowest threshold cycle (Ct) and the highest cycle-to-cycle increase of each specific fluorescent signal (ΔRn). After optimization, the 20 μL reaction mixture contained: 1 μL of viral DNA (100 ng) or control plasmid DNA, 10 μL of Premix Ex Taq (5 U/μL), 0.8 μL of each pair of primers (10 μM) (pp38-F/R were 1.6 μL), 0.4 μL of each probe (10 μM). The amplification condition was 95°C for 30s, followed by 40 cycles of 95°C for 5 s and 60°C for 30 s. The fluorescence signal was determined at the end of each cycle of the 60°C extension step. Each sample was detected by the quadruplex real-time PCR in triplicate and the Ct value was determined using the log phase of each reaction.

### Specificity, sensitivity, repeatability of the quadruplex real-time PCR

To assess the specificity of the real-time PCR assay, DNA from three species of MDV (RB1B, CVI988, HVT), cDNA/DNA from other viruses (ALV, REV, CIAV, AIV, NDV, FAV, IBDV) and DNA from CEF (negative control) were tested. The plasmids from 10^4^ to 10^0^ copies/μL were used as templates for amplification to determine the limit of detection (LOD) of each plasmid in quadruplex real-time PCR assay. The sensitivity comparison between the quadruplex real-time PCR assay and the PCR assays was using three kinds of standard plasmids in 10-fold serial dilutions. The repeatability (intra-assay precision and inter-assay precision) of the real-time PCR assay for MDV was evaluated using three different concentrations (10^7^, 10^5^, and 10^3^ copies/μL) of the standard plasmid. For intra-assay variability, each dilution was detected in triplicate on the same day, while for inter-assay variability, each dilution was tested in three independent experiments performed by two operators on different days under MIQE guidelines (32). The coefficients of variation of the Ct values were calculated based on the intra-assay or inter-assay results.

### Animal experiment

A total of 20 one-day-old specific pathogen-free (SPF) White Leghorn chickens purchased from Beijing Vital River Laboratory Animal Technology Co., Ltd. (Beijing, China) were randomly divided into four groups: control group, challenged group, vaccinated group, and vaccinated/challenged group, with 5 chickens in each group. All procedures were performed under the license of Yangzhou University. SPF chickens were reared in animal isolators and provided with sterilized feed and water. Chickens in the vaccinated and the vaccinated/challenged groups were immunized subcutaneously with 3000 pfu CVI988 in 200 μL at 1 day of age. At 6 days of age, chickens in the challenged and the vaccinated/challenged groups were infected intra-abdominally with 1000 pfu MD5 in 200 μL. Chickens in the control group were injected with an equal volume of DMEM at the same time. Viral DNA was extracted from feather samples using Axyprep Multisource Total DNA Miniprep Kit following the manufacturer’s instructions. Viral DNA was eluted with 50 μL of nuclease-free double distilled water (ddH_2_O) and stored at −80°C until use. Each of the chicken feather tip DNA samples was analyzed using the quadruplex real-time PCR. The appropriate standard curves were included in every run and were used to quantify viral copies per 10^6^ cells in each sample.

### Clinical sample detection

To further evaluate the quadruplex real-time PCR assay, the 112 clinical samples from 5 flocks were detected by the quadruplex real-time PCR assay. Afterwards, the virulent MDV-positive samples identified by quadruplex real-time PCR assay were tested for the identification of 814 strain and virulent MDV by *meq* gene PCR.

### PCR amplification of MDV *meq* gene

The primers used to amplify the *meq* gene of MDV are shown in Table 1. DNA from RB1B and CVI988 were used as PCR positive controls. PCR products were analyzed by electrophoresis and visualized by gelsafe staining (YPHbio, Beijing, China) on 1% agarose gels.

### Statistical analysis

The number of the MDV genome per million cells from the collected feathers were normalized using the following formula: normalized MDV viral load = (MDV genome copy number/chicken genome copy number) × 10^6^ (31). The standard curves of the singleplex and quadruplex real-time PCR assays and the repeatability of the quadruplex real-time PCR assay were plotted using GraphPad Prism (Version 8.0.1) and expressed as mean ± SD or mean ± 95%CI. The Student’s *t*-test was used to assess the differences between two groups. Results were considered to be statistically significant when *p < 0.05, **p < 0.01, and ***p < 0.001.

## Results

### Optimized quadruplex real-time PCR assay

**Figure 1:**
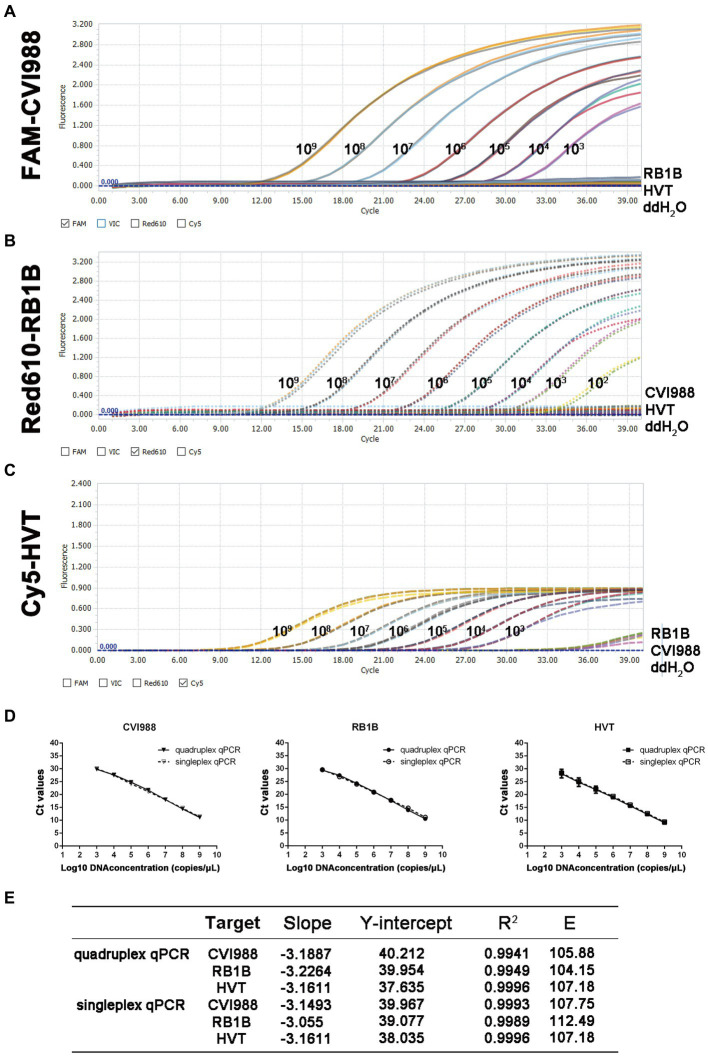
Standard curve and sensitivity tests for quadruplex real-time PCR assay of MDV. Sensitivity of quadruplex real-time PCR assay **(A)** pGEMT-pp38-CVI988, **(B)** pGEMT-pp38-RB1B and **(C)** pGEMT-sorf1-HVT. 10-fold serial dilutions of the DNA plasmid were used to perform the real-time PCR to obtain the expanded curve of the assay. **(D)** Establishment of the standard curve for quadruplex and singleplex real-time PCR assay. The 10-fold serial dilutions ranging from 1.0 × 10^9^ to 1.0 × 10^3^ copies/μL of DNA plasmid were tested in the real-time PCR. Each point corresponds to the mean ± 95% Confidence intervals of three replicates. The horizontal axis displays the log10 number of plasmid copies. The vertical axis displays the Ct values from the singleplex and quadruplex real-time PCR assays for each virus. **(E)** The slope, Y-intercept, correlation coefficient R^2^ and amplification efficiency of quadruplex and singleplex real-time PCR assay.

In the quadruplex real-time PCR, the four types of TaqMan probes were labeled with different fluorophores (FAM, Red610, Cy5, and Vic) for each virus and *ovo* gene to ensure the normal recognition of the instrument. The standard curves of the quadruplex real-time PCR were generated using 10-fold dilution series ranging from 10^9^ to 10^3^ copies of the standard plasmids per reaction (Figures 1A–D). The results showed that quadruplex real-time PCR could efficiently detect all target genes of the four kinds of standard positive plasmids with high correlation values. The corresponding standard curves and correlation coefficients were *y* = −3.1887x + 40.212 (*R*^2^ = 0.9941; *E* = 105.88) for CVI988, *y* = −3.2264x + 39.985 (*R*^2^ = 0.9949; *E* = 104.15) for RB1B and *y* = −3.1611x + 37.635 (*R*^2^ = 0.9996; *E* = 107.18) for HVT as shown in Figure 1E. Furthermore, no significant difference was observed between the amplification efficiencies of the quadruplex assay and the singleplex assays for each fluorescently-labeled probe (*p* > 0.05). The results demonstrated the high efficiency of the quadruplex real-time PCR assay.

### Specificity of the quadruplex real-time PCR assay

**Figure 2:**
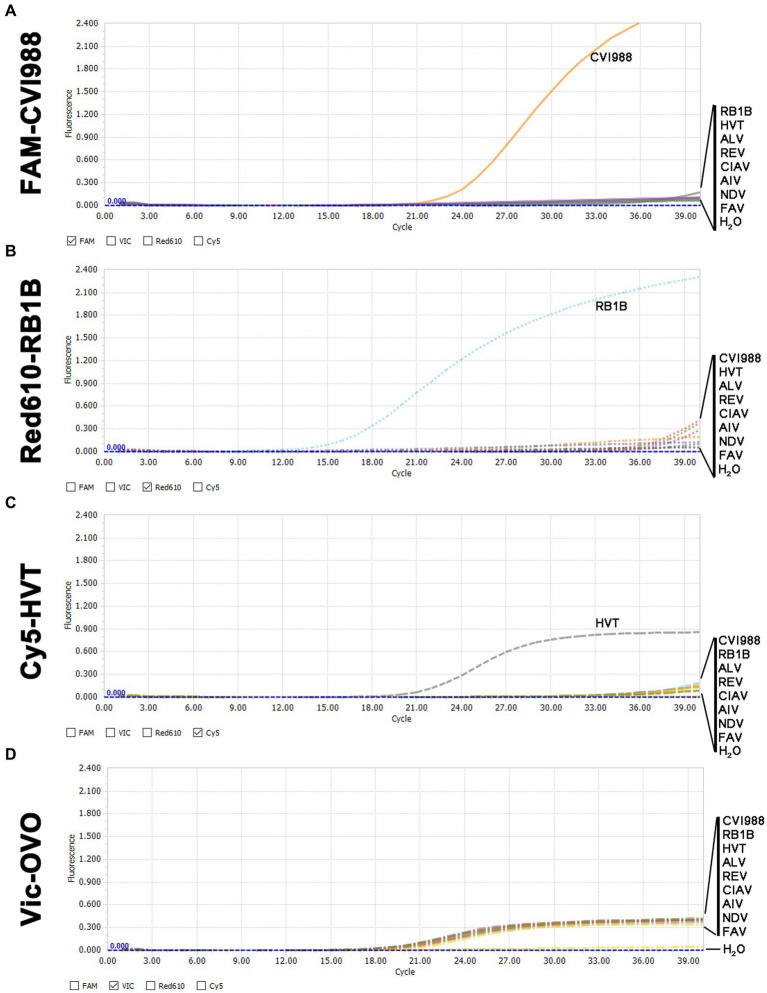
Specificity analysis of the quadruplex real-time PCR assay. **(A)** Only CVI988 showed a positive fluorescence signal, and no positive signal was observed with other viruses in the FAM-channel. **(B)** Only RB1B showed a positive fluorescence signal, and no positive signal was observed with other viruses in the Red610-channel. **(C)** Only HVT showed a positive fluorescence signal, and no positive signal was observed with other viruses in the Cy5-channel. **(D)** All DNA samples for internal control showed a positive fluorescence signal in the VIC-channel.

In the quadruplex real-time PCR, when RB1B, CVI988, and HVT positive DNA samples were used as templates, only the corresponding FAM (Figure 2A), Red610 (Figure 2B), and Cy5 (Figure 2C) fluorescent signals could be specifically detected, respectively, and VIC fluorescent signals could be detected in all samples (Figure 2D). No positive fluorescence signal of FAM, Red610 and Cy5 was obtained when other viruses (ALV, REV, CIAV, AIV, NDV, FAV) were tested (Figure 2). These results supported the high specificity of the new quadruplex real-time PCR assay.

### Sensitivity of the quadruplex real-time PCR assay

**Figure 3:**
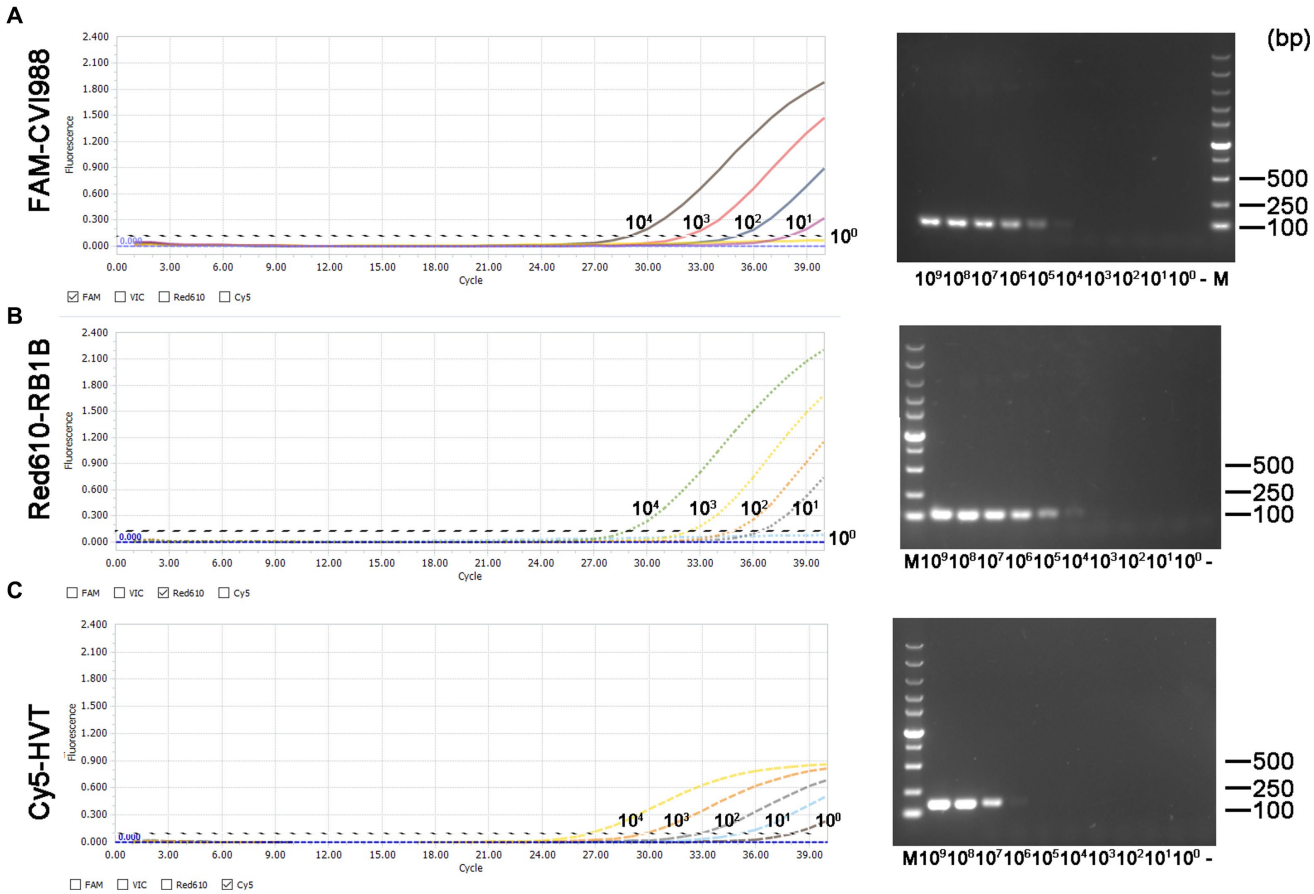
Sensitivity comparison between the quadruplex real-time PCR assay (left) and the PCR assays (right) using three kinds of the standard plasmids in 10-fold serial dilutions. **(A)** pGEMT-pp38-CVI988, **(B)** pGEMT-pp38-RB1B, and **(C)** pGEMT-sorf1-HVT.

The minimum effective positive fluorescence (EPF) was set at 0.1 according to the instrument’s default settings. The Ct value was recorded when the EPF was 0.1, and Ct values (FAM-and Red610-channels) >37 were considered CVI988- and virulent MDV-negative, while Ct values (Cy5-channel) >35 were considered HVT-negative (33). By using 10-fold serial dilutions of the standard plasmid, the LODs of CVI988, HVT and virulent MDV DNA molecules of the quadruplex real-time PCR were determined to be 10^1^ copies/μL (Figure 3 left). However, the LODs of the PCR assays were determined to be 10^4^ copies/μL for CVI988 and virulent MDV (Figures 3A,B right) and 10^6^ copies/μL for HVT (Figure 3C right). The results showed that the new assay had 1000 times lower detection limit when compared with PCR assays, indicating that the established real-time PCR was more sensitive than PCR assays.

### Repeatability of the quadruplex real-time PCR assay

The repeatability of the quadruplex real-time PCR assay was evaluated by detecting different concentrations of the standard plasmid. The intra-assay and inter-assay coefficients of variation (CVs) of Ct values for the quadruplex real-time PCR assay ranged from 0.046 to 2.465% and 0.114 to 1.869%, respectively (Supplementary Table 1).

### Dynamics of two kinds of MD viruses in chicken feather tips

**Figure 4:**
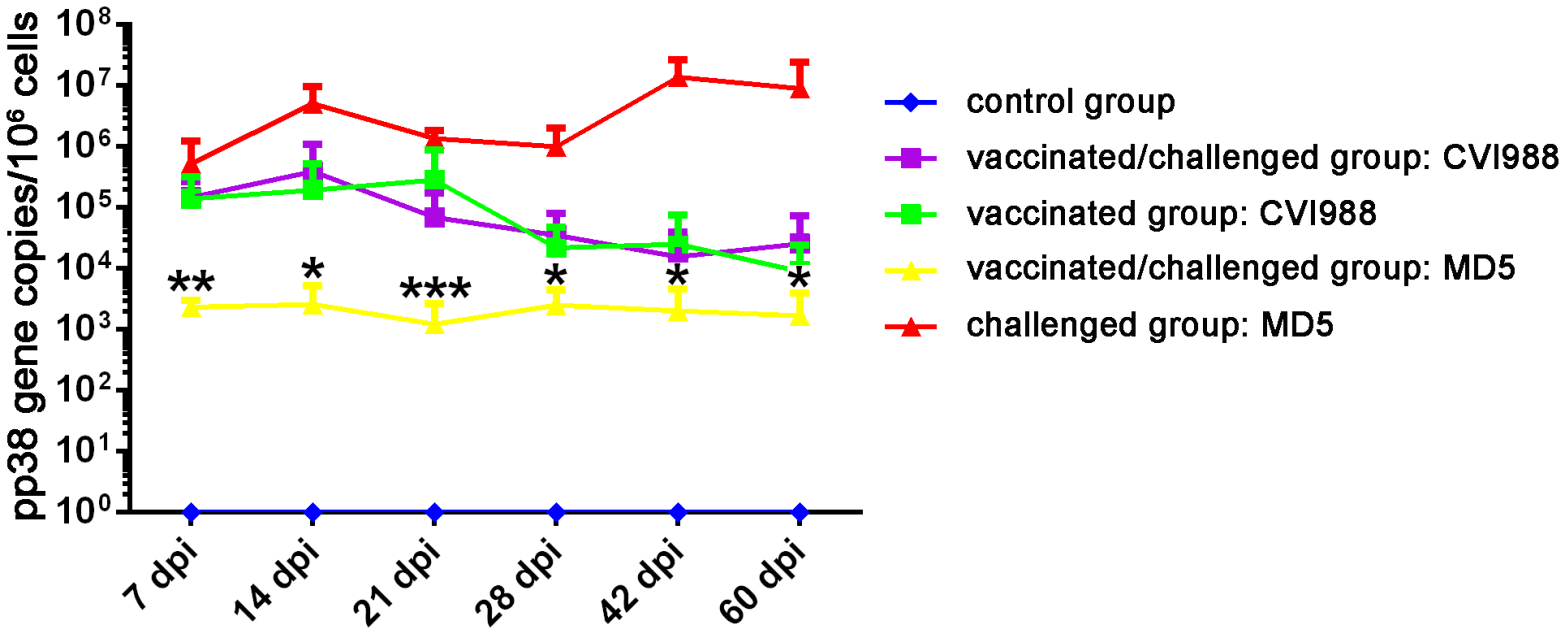
The viral replication dynamics of CVI988 and MD5 in the feather tips of the birds by quadruplex real-time PCR assay. The results were presented as mean ± SD. *, **, and *** indicated a significant difference in MD5 viral load between the challenged and vaccinated/challenged groups. *p < 0.05; **p < 0.01, and ***p < 0.001.

To further evaluate the quadruplex real-time PCR assay, feather tip samples from the artificially infected animal experiment were tested. The viral copy levels of CVI988 and MD5 at various time-points post-infection in feather tips in the four groups were shown in Figure 4. The morbidity rate of 88.89% (16/18) and mortality rate of 72.22% (13/18) were observed only in the group challenged with MD5. Although the chickens were immunized with CVI988 at a higher dose than MD5, the viral copies of MD5 in the challenged group were much higher than those of CVI988 in the vaccinated group. The MD5 genome was quantifiable from 7 dpi onwards and peaked at 14 dpi with almost 10^7^ copies/10^6^ cells, followed by a decrease in genome load toward 21 dpi, afterwards, the viral load of MD5 continued to increase reaching 10^7^ copies/10^6^ cells at 42 and 60 dpi. However, the CVI988 genome increased slowly to about 10^5^ copies/10^6^ cells from 7 to 21 dpi, followed by a decline in genome load of 10^4^ copies/10^6^ cells until 60 dpi. Notably, it was shown that MD5 had no significant effect on the viral copies level of CVI988 in feather tips, except for a slightly lower viral load of CVI988 in the vaccinated group compared to the vaccinated/challenged group at 21 dpi (*p* > 0.05). The viral copies of CVI988 in feather tips from the vaccinated and vaccinated/challenged groups reached to 10^5^ copies per 10^6^ cells at 7 dpi and, then fluctuated between 10^4^ and 10^5^ copies per 10^6^ cells. Nevertheless, CVI988 vaccination significantly reduced the mean level of viral copies of MD5 in feather tips (*p* < 0.05). The replication of MD5 was delayed and significantly reduced to 10^3^ copies per 10^6^ cells in feather tips in vaccinated chickens at each detection time point, whereas MD5 replication in the challenge group was 10^4^ copies at 7 dpi and peaked at 5.0 × 10^6^ copies at 14 dpi, then fluctuated between 10^6^ and 10^7^ copies per 10^6^ cells.

### Clinical application

To further evaluate the quadruplex real-time PCR assay, 112 clinical samples from 5 flocks were tested (Table 2). The background information of the feather samples were shown in Supplementary Table 3. The results showed that 5 out of 60 samples were determined as virulent-MDV positive in flock 1 (unvaccinated), 19 of 20 samples were determined as CVI988-positive in flock 2 (CVI988-vaccinated). 17 and 15 of 20 samples were determined as CVI988-positive and HVT-positive, respectively, in flock 3 (HVT + CVI988-vaccinated). 8 of 8 samples were determined as HVT-positive in flock 4 (HVT-vaccinated) but also found 1 positive for CVI988. 4 samples in flock 5 (814 strain-vaccinated) were found positive for virulent-MDV but were further determined as vaccine strain by PCR (Table 3 and Supplementary Figure 1), sequencing results showed that these samples in flock 5 have the same *meq* gene as 814 strain.

**Table 2:** Clinical application of the quadruplex real-time PCR assay.

Method	Targets	Flock 1 (not vaccinated)	Flock 2 (CVI988-vaccinated)	Flock 3 (HVT + CVI988-vaccinated)	Flock 4 (HVT-vaccinated)	Flock 5 (814 strain-vaccinated)
Quadruplex real-time PCR assay	CVI988	0/60	19/20	17/20	1/8	0/4
	Virulent MDV	5/60*	0/20	0/20	0/8	4/4*
	HVT	0/60	0/20	15/20	8/8	0/4

112 clinical samples from 5 flocks were tested and the number of positive samples were shown, respectively. *Means the samples determined as virulent MDV-positive by quadruplex real-time PCR assay were used for PCR verification.

**Table 3:** Application of *meq* gene PCR for verification of virulent MDV.

Method	Targets	Flock 1 (not vaccinated)	Flock 5 (814 strain-vaccinated)
Verification of virulent MDV	Virulent MDV	3/5	0/4
	Vaccine MDV	0/5	4/4

The samples determined as virulent MDV-positive by quadruplex real-time PCR assay in flock 1 and flock 5 were tested and the number of positive samples were shown, respectively.

## Discussion

Many vaccines have been used for the prevention and control of MD, including the attenuated vaccines (CVI988 and 814 strain), HVT (18, 34, 35), and especially the *meq*-deleted MDV-1 strain developed in recent years (8). These new MDV-1 vaccine viruses made it difficult to distinguish from virulent MDV. How to evaluate the immunization status of the vaccine and how to make a rapid and reliable diagnosis of MD are urgent problems that need to be addressed. In this study, a quadruplex real-time PCR assay based on TaqMan probes combined with PCR can effectively differentiate and accurately quantify HVT, CVI988 and virulent MDV-1, and can also be applied for the rapid detection of field MDV infection in poultry farms immunized with 814 or *meq*-deleted MDV strains. This assay showed advantages over others due to its ability to differentiate and quantify CVI988, HVT and virulent MDV in one reaction. No interference between the fluorescence signals of the probes was observed in this assay and different fluorescent probes have good specificity and noise is very low.

To further analyze the practicality of this assay, chicken feather tip samples collected from five chicken flocks and animal experiments were detected. The results showed that the quadruplex real-time PCR had a higher positive detection rate for three species of MDV strains compared with PCR assays. In agreement with a previous study (36), the MD5 genome was quantifiable from 7 dpi and peaked at 14 dpi, followed by a decline in viral load to 21 dpi in the challenged and vaccinated/challenged groups. However, CVI988 genome increased slightly at 14 dpi but was not significantly different between the vaccinated and vaccinated/challenged groups (*p* > 0.05). CVI988 vaccine can inhibit the replication of virulent MDV in feathers, while MD5 challenge had no significant effect on the replication of CVI988 in feathers.

The results of samples collected from field cases showed that the 4 samples from flock 5 were immunized with the 814 strain, but were misjudged as virulent MDV-positive by the quadruplex real-time PCR assay (Supplementary Table 2). Our method is to combine the *meq* gene PCR with the quadruplex real-time PCR to identify virulent MDV from flocks immunized with strain 814, SC9-1 and other MDV-1 vaccine viruses. In addition, the results of clinical samples showed that vaccination could reduce the positive rate of virulent MDV compared with unvaccinated flocks (Table 2). Interestingly, one sample was detected as CVI988-positive in flock 4, possibly because flock 4 was previously vaccinated with CVI988 and chickens were infected with residual CVI988 from the environment. In addition, gallid herpesvirus 3 (SB-1) has very low homology with other MDV serotype strains according to the complete genome (37). Theoretically, this method is not suitable for the detection of the SB-1 strain.

In summary, we developed a quadruplex real-time PCR assay to differentiate between the vaccine and virulent MDV strains. This method has the advantages of being reliable, sensitive, specific, and accurate in the differential quantification of CVI988, HVT, and virulent MDVs, confirming the success of vaccination and monitoring the circulation of virulent field strains. The virulent MDV from flocks immunized with strain 814 or SC9-1 could be identified by quadruplex real-time PCR combined with PCR.

## Data availability statement

The original contributions presented in the study are included in the article/Supplementary material, further inquiries can be directed to the corresponding author.

## Ethics statement

The animal study was reviewed and approved by the Animal Ethics Committee of Yangzhou University.

## Author contributions

SW and AQ wrote the manuscript. SW and TD performed the experiment and data analysis. SW, AQ, JY, KQ, and HS designed the study. All authors contributed to the article and approved the submitted version.

## Funding

This project was supported by the National Science Foundation of China (31972717 and 31761133002), the Priority Academic Program Development of Jiangsu Higher Education Institutions, the Jiangsu Co-Innovation Center for the Prevention and Control of Important Animal Infectious Diseases and Zoonoses. SW was supported by the Research Innovation Project of Jiangsu Province Graduate Students (XSJCX20-033). The funding bodies did not play direct roles in the design of the study and collection, analysis, or interpretation of data or in writing the manuscript.

## Conflict of interest

The authors declare that the research was conducted in the absence of any commercial or financial relationships that could be construed as a potential conflict of interest.

## Publisher’s note

All claims expressed in this article are solely those of the authors and do not necessarily represent those of their affiliated organizations, or those of the publisher, the editors and the reviewers. Any product that may be evaluated in this article, or claim that may be made by its manufacturer, is not guaranteed or endorsed by the publisher.

## Supplementary material

The Supplementary material for this article can be found online at: https://www.frontiersin.org/articles/10.3389/fvets.2023.1161441/full#supplementary-material

SUPPLEMENT TABLE 1 Intra-repeatability and inter-reproducibility of the quadruplex real-time PCR assay. The repeatability and reproducibility were shown in mean ± standard deviation (SD) from three replicates.

SUPPLEMENT TABLE 2 The detailed information of the detected clinical samples includes flock number and location, sample number, CT value of fluorescence quantification, and corresponding virus copy number.

SUPPLEMENT TABLE 3 The background information of the feather samples from 5 flocks.

SUPPLEMENT FIGURE 1 Meq gene amplified by PCR. A1-A4: 4 samples from flock 5; R: RB1B DNA; C: CVI988 DNA; -: negative control; M: DNA marker.
