# Supplementary material for: A quadruplex real-time PCR assay combined with a conventional PCR for the differential detection of Marek’s disease virus vaccines and field strains
Source: Front Vet Sci. 2023 May 12;10:1161441. doi: 10.3389/fvets.2023.1161441 (PMC10213282; doi:10.3389/fvets.2023.1161441)
Supplement: SUPPLEMENT TABLE 1 — Intra-repeatability and inter-reproducibility of the quadruplex real-time PCR assay. The repeatability and reproducibility were shown in mean ± standard deviation (SD) from three replicates. [file Table_1.docx]

**Supplement Table 1.** Intra-repeatability and inter-reproducibility of the quadruplex real-time PCR assay. The repeatability and reproducibility were shown in mean ± standard deviation (SD) from three replicates.

| **Target** | **Standard sample(lg (copies/μL))** | **Intra reproductivity** | **Standard deviation(SD)** | **Coefficients of variation(%)** | **Inter reproductivity** | **SD** | **Coefficients of variation(%)** |
| --- | --- | --- | --- | --- | --- | --- | --- |
| **FAM-CVI988** | 7 | 18.057 | 0.012 | 0.064 | 18.073 | 0.023 | 0.129 |
|  | 5 | 24.773 | 0.159 | 0.644 | 25.314 | 0.473 | 1.869 |
|  | 3 | 29.807 | 0.215 | 0.723 | 29.374 | 0.374 | 1.275 |
| **Red610-RB1B** | 7 | 17.570 | 0.044 | 0.248 | 17.484 | 0.101 | 0.576 |
|  | 5 | 24.273 | 0.110 | 0.454 | 24.228 | 0.060 | 0.247 |
|  | 3 | 29.420 | 0.044 | 0.148 | 29.388 | 0.071 | 0.241 |
| **Cy5-HVT** | 7 | 15.577 | 0.038 | 0.243 | 15.499 | 0.068 | 0.439 |
|  | 5 | 21.903 | 0.540 | 2.465 | 21.856 | 0.044 | 0.201 |
|  | 3 | 28.127 | 0.680 | 2.416 | 28.142 | 0.081 | 0.288 |
| **Vic-ovo** | 7 | 17.600 | 0.085 | 0.482 | 17.598 | 0.020 | 0.114 |
|  | 5 | 23.790 | 0.226 | 0.951 | 23.753 | 0.032 | 0.135 |
|  | 3 | 30.670 | 0.014 | 0.046 | 30.607 | 0.055 | 0.181 |
