# Supplementary material for: A quadruplex real-time PCR assay combined with a conventional PCR for the differential detection of Marek’s disease virus vaccines and field strains
Source: Front Vet Sci. 2023 May 12;10:1161441. doi: 10.3389/fvets.2023.1161441 (PMC10213282; doi:10.3389/fvets.2023.1161441)
Supplement: SUPPLEMENT TABLE 2 — The detailed information of the detected clinical samples includes flock number and location, sample number, CT value of fluorescence quantification, and corresponding virus copy number. [file Table_2.docx]

**Supplement Table 2.** The detailed information of the detected clinical samples includes flock number and location, sample number, CT value of fluorescence quantification, and corresponding virus copy number.

| **Flock 1 from Tangshan city (no vaccinated)** | | | | | | | |
| --- | --- | --- | --- | --- | --- | --- | --- |
| **samples** | **Ct (VIC-OVO)** | **Ct(FAM-CVI)** | **CVI988(copy/10^6^ cells)** | **Ct(Red610-VIR)** | **virulent MDV(copy/10^6^ cells)** | **Ct(Cy5-HVT)** | **HVT(copy/10^6^ cells)** |
| A1 | 19.81 | - | - | - | - | - | - |
| A2 | 19.22 | - | - | - | - | - | - |
| A3 | 20.13 | - | - | - | - | - | - |
| A4 | 19.26 | - | - | - | - | - | - |
| A5 | 19.66 | - | - | - | - | - | - |
| A6 | 21.04 | - | - | - | - | - | - |
| A7 | 19.81 | - | - | - | - | - | - |
| A8 | 20.4 | - | - | - | - | - | - |
| A9 | 20.77 | - | - | - | - | - | - |
| A10 | 19.29 | - | - | - | - | - | - |
| A11 | 21.68 | - | - | - | - | - | - |
| A12 | 19.79 | - | - | - | - | - | - |
| A13 | 21.97 | - | - | - | - | - | - |
| A14 | 22.11 | - | - | - | - | - | - |
| A15 | 21.63 | - | - | - | - | - | - |
| A16 | 21.46 | - | - | - | - | - | - |
| A17 | 20.58 | - | - | - | - | - | - |
| A18 | 21.37 | - | - | - | - | - | - |
| A19 | 21.08 | - | - | - | - | - | - |
| A20 | 20.47 | - | - | - | - | - | - |
| A21 | 21.38 | - | - | - | - | - | - |
| A22 | 21.68 | - | - | - | - | - | - |
| A23 | 22.25 | - | - | - | - | - | - |
| A24 | 22.66 | - | - | - | - | - | - |
| A25 | 22.71 | - | - | - | - | - | - |
| A26 | 22.79 | - | - | - | - | - | - |
| A27 | 22.88 | - | - | - | - | - | - |
| A28 | 21.3 | - | - | - | - | - | - |
| A29 | 21.03 | - | - | - | - | - | - |
| A30 | 20.45 | - | - | - | - | - | - |
| B1 | 21.38 | - | - | - | - | - | - |
| B2 | 19.03 | - | - | 20.1 | 100748 | - | - |
| B3 | 20.89 | - | - | - | - | - | - |
| B4 | 19.47 | - | - | - | - | - | - |
| B5 | 20.77 | - | - | - | - | - | - |
| B6 | 18.73 | - | - | - | - | - | - |
| B7 | 19.18 | - | - | - | - | - | - |
| B8 | 19.53 | - | - | 22.77 | 21145 | - | - |
| B9 | 20.93 | - | - | - | - | - | - |
| B10 | 20.22 | - | - | - | - | - | - |
| B11 | 24.43 | - | - | - | - | - | - |
| B12 | 24.77 | - | - | - | - | - | - |
| B13 | 25.99 | - | - | - | - | - | - |
| B14 | 25.27 | - | - | - | - | - | - |
| B15 | 22.76 | - | - | - | - | - | - |
| B16 | 21.96 | - | - | - | - | - | - |
| B17 | 23.99 | - | - | - | - | - | - |
| B18 | 21.34 | - | - | - | - | - | - |
| B19 | 21.38 | - | - | - | - | - | - |
| B20 | 24.58 | - | - | 22.18 | 1043139 | - | - |
| B21 | 21.07 | - | - | - | - | - | - |
| B22 | 23.17 | - | - | - | - | - | - |
| B23 | 22.01 | - | - | - | - | - | - |
| B24 | 22.35 | - | - | - | - | - | - |
| B25 | 20.43 | - | - | - | - | - | - |
| B26 | 24.64 | - | - | 28.92 | 8857 | - | - |
| B27 | 24.23 | - | - | - | - | - | - |
| B28 | 25.09 | - | - | - | - | - | - |
| B29 | 22.26 | - | - | 22.92 | 124498 | - | - |
| B30 | 23.8 | - | - | - | - | - | - |
| RB1B | 24.57 | - | - | 16.69 | 781703409 | - | - |
| CVI988 | 24.92 | 17.66 | 759445171 | - | - | - | - |
| HVT | 23.86 | - | - | - | - | 22.47 | 45660816 |
| CEF | 22.23 | - | - | - | - | - | - |
| H_2_O | - | - | - | - | - | - | - |
| **Flock 2 from Dalian city(CVI988-vaccinated)** | | | | | | | |
| **samples** | **Ct (VIC-OVO)** | **Ct(FAM-CVI)** | **CVI988(copy/10^6^ cells)** | **Ct(Red610-VIR)** | **virulent MDV(copy/10^6^ cells)** | **Ct(Cy5-HVT)** | **HVT(copy/10^6^ cells)** |
| B1 | 24.53 | 20.64 | 4289307 | - | - | - | - |
| B2 | 22.53 | 18.73 | 4297892 | - | - | - | - |
| B3 | 24.21 | 21.4 | 1987678 | - | - | - | - |
| B4 | 22.19 | - | - | - | - | - | - |
| B5 | 22.58 | 22.67 | 258580 | - | - | - | - |
| B6 | 21.59 | 27.42 | 4235 | - | - | - | - |
| B7 | 22.92 | 29.6 | 2193 | - | - | - | - |
| B8 | 21.15 | 23.32 | 60408 | - | - | - | - |
| B9 | 24.6 | 33.18 | 526 | - | - | - | - |
| B10 | 21.86 | 20.93 | 553284 | - | - | - | - |
| B11 | 25.99 | 28.35 | 44782 | - | - | - | - |
| B12 | 24.52 | 29 | 10177 | - | - | - | - |
| B13 | 20.79 | 29.25 | 651 | - | - | - | - |
| B14 | 21.75 | 31.86 | 192 | - | - | - | - |
| B15 | 22.53 | 30.83 | 690 | - | - | - | - |
| B16 | 21.52 | 27.01 | 5427 | - | - | - | - |
| B17 | 21.95 | 24.15 | 57550 | - | - | - | - |
| B18 | 19.74 | 19.82 | 286446 | - | - | - | - |
| B19 | 20.98 | 18.84 | 1365256 | - | - | - | - |
| B20 | 21.21 | 26.64 | 5726 | - | - | - | - |
| RB1B | 24.34 | - | - | 14.58 | 200509046 | - | - |
| CVI988 | 24.34 | 19.66 | 7636614 | - | - | - | - |
| HVT | 24.34 | - | - | - | - | 22.37 | 184781 |
| CEF | 24.34 | - | - | - | - | - | - |
| H_2_O | - | - | - | - | - | - | - |
| **Flock 3 from Yinchuan city (HVT+CVI988 vaccinated)** | | | | | | | |
| **samples** | **Ct (VIC-OVO)** | **Ct(FAM-CVI)** | **CVI988(copy/10^6^ cells)** | **Ct(Red610-VIR)** | **virulent MDV(copy/10^6^ cells)** | **Ct(Cy5-HVT)** | **HVT(copy/10^6^ cells)** |
| A2 | 23.93 | 29.37 | 5190 | - | - | - | - |
| A4 | 22.89 | 23.86 | 135554 | - | - | - | - |
| A8 | 22.02 | 31.16 | 382 | - | - | 31.04 | 68 |
| B12 | 22.95 | 20.41 | 1706085 | - | - | - | - |
| B17 | 24.52 | 33.4 | 424 | - | - | 33.87 | 48 |
| D1 | 23.95 | 28.86 | 7605 | - | - | - | - |
| D2 | 23.01 | 34.53 | 66 | - | - | 26.28 | 4285 |
| D3 | 22.36 | 23.51 | 121163 | - | - | 20.21 | 227941 |
| D4 | 23.07 | 23.48 | 201889 | - | - | - | - |
| D8 | 23.65 | 34.73 | 89 | - | - | 32.69 | 62 |
| D9 | 24.96 | 33.52 | 527 | - | - | 33.01 | 122 |
| D10 | 24.25 | - | - | - | - | 24.97 | 26136 |
| D11 | 23.15 | 24.23 | 124117 | - | - | 23.32 | 40761 |
| D13 | 23.62 | - | - | - | - | 24.46 | 24557 |
| D14 | 23 | 33.92 | 102 | - | - | 31.31 | 109 |
| D15 | 22.57 | - | - | - | - | 21.1 | 137745 |
| D16 | 23.11 | 33.43 | 157 | - | - | 33.02 | 34 |
| E1 | 23.37 | 25.97 | 41110 | - | - | 25.24 | 11713 |
| E2 | 22.64 | 31.58 | 433 | - | - | 31.41 | 79 |
| E7 | 24.07 | 34.27 | 166 | - | - | 33.16 | 59 |
| RB1B | 21.63 | - | - | 17.2 | 4782205 | - | - |
| CVI988 | 21.63 | 17.99 | 3946118 | - | - | - | - |
| HVT | 21.63 | - | - | - | - | 19.09 | 311756 |
| CEF | 23.12 | - | - | - | - | - | - |
| H_2_O | - | - | - | - | - | - | - |
| **Flock 4 from Lishui city (HVT-vaccinated)** | | | | | | | |
| **samples** | **Ct (VIC-OVO)** | **Ct(FAM-CVI)** | **CVI988(copy/10^6^ cells)** | **Ct(Red610-VIR)** | **virulent MDV(copy/10^6^ cells)** | **Ct(Cy5-HVT)** | **HVT(copy/10^6^ cells)** |
| B1 | 22.56 | - | - | - | - | 21.69 | 194476.72 |
| B2 | 24.24 | - | - | - | - | 33.37 | 157.15 |
| B3 | 23.39 | - | - | - | - | 22.26 | 254770.04 |
| B4 | 23.06 | - | - | - | - | 33.46 | 55.56 |
| C1 | 21.25 | - | - | - | - | 24.51 | 8454.16 |
| C2 | 21.55 | 32.2 | 248.44 | - | - | 27.2 | 1526.39 |
| C3 | 21.76 | - | - | - | - | 28.88 | 533.95 |
| C4 | 22.01 | - | - | - | - | 20.42 | 311475.01 |
| RB1B | 27.85 | - | - | 17.92 | 934884611.7 | - | - |
| CVI988 | 27.85 | 19.15 | 558051411 | - | - | - | - |
| HVT | 27.85 | - | - | - | - | 23.47 | 4192853.44 |
| CEF | 27.85 | - | - | - | - | - | - |
| H_2_O | - | - | - | - | - | - | - |
| **Flock 5 from Lishui city (814 strain-vaccinated)** | | | | | | | |
| **samples** | **Ct (VIC-OVO)** | **Ct(FAM-CVI)** | **CVI988(copy/10^6^ cells)** | **Ct(Red610-VIR)** | **virulent MDV(copy/10^6^ cells)** | **Ct(Cy5-HVT)** | **HVT(copy/10^6^ cells)** |
| A1 | 22.11 | - | - | 17.91 | 8237057.65 | - | - |
| A2 | 23.13 | - | - | 22.49 | 727705.71 | - | - |
| A3 | 23.1 | - | - | 26.03 | 56754.61 | - | - |
| A4 | 22.75 | - | - | 21.23 | 1306875.97 | - | - |
| RB1B | 27.85 | - | - | 17.92 | 934884611.7 | - | - |
| CVI988 | 27.85 | 19.15 | 558051411 | - | - | - | - |
| HVT | 27.85 | - | - | - | - | 23.47 | 4192853.44 |
| CEF | 27.85 | - | - | - | - | - | - |
| H_2_O | - | - | - | - | - | - | - |
