# Supplementary material for: A quadruplex real-time PCR assay combined with a conventional PCR for the differential detection of Marek’s disease virus vaccines and field strains
Source: Front Vet Sci. 2023 May 12;10:1161441. doi: 10.3389/fvets.2023.1161441 (PMC10213282; doi:10.3389/fvets.2023.1161441)
Supplement: SUPPLEMENT TABLE 3 — The background information of the feather samples from 5 flocks. [file Table_3.docx]

**Supplement Table 3.** The background information of the feather samples from 5 flocks.

| **Flocks** | **No. of samples** | **Chicken breed** | **Type of vaccine** | **Vaccine age (day)** | **Sampling age (day)** |
| --- | --- | --- | --- | --- | --- |
| Flock 1 | 60 | white feather broiler | - | - | 35 |
| Flock 2 | 20 | unknown | CVI988 | 1 | 21 |
| Flock 3 | 20 | layer | CVI988+HVT | 1 | 28 |
| Flock 4 | 8 | Aijiaohuang | HVT | 1 | 29 |
| Flock 5 | 4 | Tianluma | 814 strain | 1 | 29 |
