# Supplementary figures and images for: A quadruplex real-time PCR assay combined with a conventional PCR for the differential detection of Marek’s disease virus vaccines and field strains
Source: Front Vet Sci. 2023 May 12;10:1161441. doi: 10.3389/fvets.2023.1161441 (PMC10213282; doi:10.3389/fvets.2023.1161441)

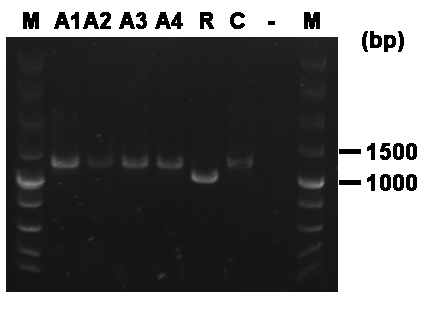

Supplement: SUPPLEMENT FIGURE 1 — Meq gene amplified by PCR. A1-A4: 4 samples from flock 5; R: RB1B DNA; C: CVI988 DNA; -: negative control; M: DNA marker. [file Image_1.tif]
